# Effects of birch encroachment, water table and vegetation on methane emissions from peatland microforms in a rewetted bog

**DOI:** 10.1038/s41598-024-52349-0

**Published:** 2024-01-30

**Authors:** Carla Welpelo, Maren Dubbert, Bärbel Tiemeyer, Claas Voigt, Arndt Piayda

**Affiliations:** 1 Thünen Institute of Climate-Smart Agriculture, Bundesallee 65, 38116 Braunschweig, Germany; 2 Leibniz Centre for Agricultural Landscape Research (ZALF), Eberswalder Straße 84, 15374 Müncheberg, Germany

**Keywords:** Carbon cycle, Carbon cycle

## Abstract

This study investigated the influence of vegetation and microforms on methane (CH_4_) balances of a rewetted bog in north-west Germany. The two study sites are in close proximity on the same former peat extraction area, one dominated by *Sphagnum-*mosses and the other one by a dense *Betula pubescens* stand with a high *Eriophorum vaginatum* cover. The contribution of microforms (hummocks/hollows) to CH_4_ emissions and the effect of *Betula* encroachment has been studied. Transparent and opaque chambers were used to measure CH_4_ fluxes every 3–4 weeks during daytime for one year. For the estimation of annual balances, three methods were compared and the method using water level and soil temperature as explanatory variables was selected. Fluxes were scaled to the site level. The annual emissions per site are and 7.1 ± 1.5 g CH_4_-C m^−2^ year^−1^ at the treed site and 36.1 ± 3.5 g CH_4_-C m^−2^ year^−1^ at the open site, mainly controlled by higher water levels. Highest annual emissions originated from hollows at the open site, but in the vegetation period, hummock emissions tend to be higher. At the tree site, emission differences between the microforms were less pronounced. There were no differences between fluxes from transparent and opaque chambers.

## Introduction

Peatlands store approximately 547 (473–621) Gt carbon (C) on over 4 Mio. km^2^ globally^1,2^. In Europe they cover more than 5% of the land surface, making them important stocks of C. Nowadays, anthropogenic impacts such as peat extraction or drainage for agriculture result in high greenhouse gas (GHG) emissions from peatlands^3^. Peat extraction for growing media or energy has been and is still common in northern Germany, which leads to a large area of destroyed peatlands^4^. In Germany, most bogs can be found in the North-West (Lower Saxony), where over 53,000 ha of bogs have been used for industrial peat extraction^5^. Usually, former extraction sites have been rewetted as shallow flooded polders. In the last decades, strong efforts were invested in revitalizing peat extraction areas for nature conservation. Presently, climate protection has become another important objective of rewetting, as this will reduce GHG emissions and may even restore the potential of peatlands to act as atmospheric C sinks^6^. The C storage in peatland ecosystems is mainly controlled by water supply^7^, making water management and availability key factors for successful rewetting.

With elevated water level (WL) in natural or rewetted bogs, methane (CH_4_) emissions are higher than in their drained counterparts. CH_4_ is the second most important GHG and is produced by methanogens in anaerobic environments^8^. The climate impact of CH_4_ is 28 times larger than of CO_2_ (100-year time horizon for CH_4_)^9^ and has often been discussed to be the major part of the GHG balance of rewetted bogs^10,11^. Nevertheless, total GHG emissions of rewetted areas are usually low compared to drained peatlands due to their low CO_2_ emissions or even C uptake^12^.

Next to water saturation and the corresponding low availability of oxygen, CH_4_ emissions might be determined by a number of abiotic and biotic factors such as soil temperature, substrate availability, leaf area index (LAI), plant productivity and multiple other factors^8,13–15^. CH_4_ can be released to the atmosphere from water-saturated peat by diffusion, and can be oxidized to CO_2_ on its way through aerated peat layers. In addition, CH_4_ can be released via ebullition, which results in sudden emission peaks with a high spatial and temporally variability^8^. The third pathway is the gas transport through aerenchymous tissue. This is mainly controlled by vegetation composition, which is the major biotic factor controlling CH_4_ emissions^16,17^.

The gas transport through aerenchymous tissue operates as a direct connection for CH_4_ from the water saturated peat to the atmosphere^18^, bypassing the unsaturated zone. It can show a strong correlation with LAI and biomass, and especially *Eriophorum* species are known to increase CH_4_-release^13,17,19–22^. Due to rooting, vascular plants can increase the presence of easy decomposable material^23^, which is then favored for CH_4_ production.

Further, conditions of high photosynthetically active radiation (PAR) and resulting plant productivity is debated to increase CH_4_ emissions by increasing aerenchymous transport as well as by supplying easily degradable photosynthates to the root zone^8^. In some field studies, a rise of CH_4_ emissions with higher PAR could be confirmed^24^, in others it was not present^25^ or thought to be a co-dependency with temperature^26^.

Methane emissions are spatially highly variable, seasonally and in quantity, due to the different emission pathways and multiple impact parameters^15^. In bogs, this is especially pronounced between hummock and hollow microforms. Environmental factors, especially water table depth or soil temperature, can differ significantly between hummocks and hollows^27^, therefore different vegetation communities are present at these microforms^28^. These small-scale differences can lead to strong differences in rates of CH_4_ emissions^27,29–31^. Quantifying total CH_4_ emissions for each microform separately can therefore be necessary to quantify ecosystem scale CH_4_ fluxes.

Natural raised bogs are dominated by peat mosses, as *Sphagnum* is the main peat-forming species group in bogs^32^. It can store many times its own dry weight in water and ensures low pH in its direct environment^33^, fostering unfavorable growth conditions for other plants. Vegetation composition in bogs alters due to multifaceted changes of environmental conditions caused by anthropogenic impacts. This is encouraging the enhanced growth of wetland graminoids such as *Eriophorum* spp. and other vascular plants^34,35^. Vegetation succession under wet conditions will first lead to *Eriophorum* and peat mosses^36^, but may result in birch encroachment under drier hydrological conditions.

The encroachment with downy birch (*Betula pubescens*) was observed in many bogs across the northern hemisphere in the last decades^37,38^. Due to root activity, tree invasion can change the composition of nutrients and dissolved organic carbon (DOC) in bog pore water as well as accelerate the decomposition of the peat due to aeration^39^. Birches are discussed to influence the WL in bogs^40,41^, which might consequently influence vegetation development as well as O_2_ saturation of the peat, but the impact of this change on CH_4_ emissions is yet unknown. Trees also contribute to CH_4_ emissions from peatlands through emissions from trunk and shoots^42,43^. Regarding the overall influence of birches on the CH_4_ emission budget, little is known^43–45^.

Strong dependences on PAR or other meteorological parameters will cause diurnal cycles of methane fluxes, which are inadequately captured by “classic” measurements with opaque chambers, a longer closure time and gas sampling. Flux measurements with transparent and opaque chambers, covering a whole year and many daily patterns with its changes in soil temperature and radiation, will significantly improve process understanding. This also opens up new opportunities to apply different methods to calculate annual balances, as various parameters can be used as predictors.

Here, we present the CH_4_ balances of two former peat extraction sites, one successfully rewetted and the other one exhibiting a dense birch encroachment. Both sites own distinct hummock-hollow microforms. The research questions addressed here focus on the one hand on ecosystem functioning: How do the microforms differ in their methane fluxes? Can the fluxes be set in relation to microform specific environmental parameters, such as vegetation, water quality or WL? Which differences between both sites emerge consequently in their annual methane balances? On the other hand, we address important methodological questions, like the difference between fluxes from transparent and opaque chambers and how reliable annual methane balances can be calculated for the investigation area and measurement period.

## Results

### Meteorological and hydrological conditions

Mean temperature in the main measurement period (used for annual balances (November 2020–October 2021)), was 10.2 °C and total precipitation was 680 mm (German Meteorological Service, Nienburg (Weser) (12 km)). Thus, precipitation was below long term mean for this area (731 mm, 1991–2020), but still high compared to the very dry years before, while temperature was higher than the long-term average (9.9 °C, 1991–2020).

Overall, mean water levels below the peat surface (WL) were both different between microforms and sites, with the hummocks having lower mean WL than the hollows (Table 1) and the tree site being drier than the open site. WL at both sites were low after a dry summer 2020, and increased from November 2020 onwards. Due to a wet winter and spring, WL at the open site went up to the surface at hollows and above in February 2021 and stayed high over the summer. Hollows were nearly constantly flooded onwards until mid-July at the open site.

**Table 1:** Areal contribution [%] of microforms, mean water level and its maximum and minimum value over the measurement period, dissolved organic carbon (DOC), pH, Electrical conductivity (EC), mean leaf area index (LAI) of *Eriophorum* spp. (for vegetation period (1st March–30th October) and maximum LAI and surface height difference within one plot for each microform. The designation ‘T’ in the microform labelling stands for the direct proximity of a tree, while ‘nT’ denotes distance to a tree.

Microform	Areal contribution ± SD [%]	Mean water level + range [m]	DOC [mg l^−1^]	pH	EC (μS cm^−1^)	Vasc. LAI (mean/max) [m^2^ m^−2^]	Surface height difference (median ± SD) [cm]
open_hollow	56.5 ± 2.2	− 0.03 (− 0.18 to 0.06)	66 ± 3	3.8	101 ± 9	0.2 (0.6)	2 ± 1.2
open_hummock	43.5 ± 2.2	− 0.13 (− 0.30 to − 0.01)	65 ± 4	3.8	104 ± 8	5.5 (13.9)	15.5 ± 7.54
tree_nT_hollow	19.3 ± 12.2	− 0.12 (− 0.30 to 0.04)	105 ± 21	3.6	132 ± 15	0.6 (1.8)	3.5 ± 2.7
tree_nT_hummock	31.2 ± 15.1	− 0.23 (− 0.42 to − 0.09)	106 ± 21	3.6	138 ± 17	4.7 (9.2)	12.2 ± 4.9
tree_T_hollow	18.8 ± 6.3	− 0.1 (− 0.29 to 0.05)	115 ± 31	3.6	140 ± 20	0.8 (2.3)	8 + 4.9
tree_T_hummock	30.7 ± 12.9	− 0.27 (− 0.45 to − 0.1)	123 ± 22	3.6	148 ± 29	2.9 (5.1)	17.8 ± 10.4

**Figure 1:**
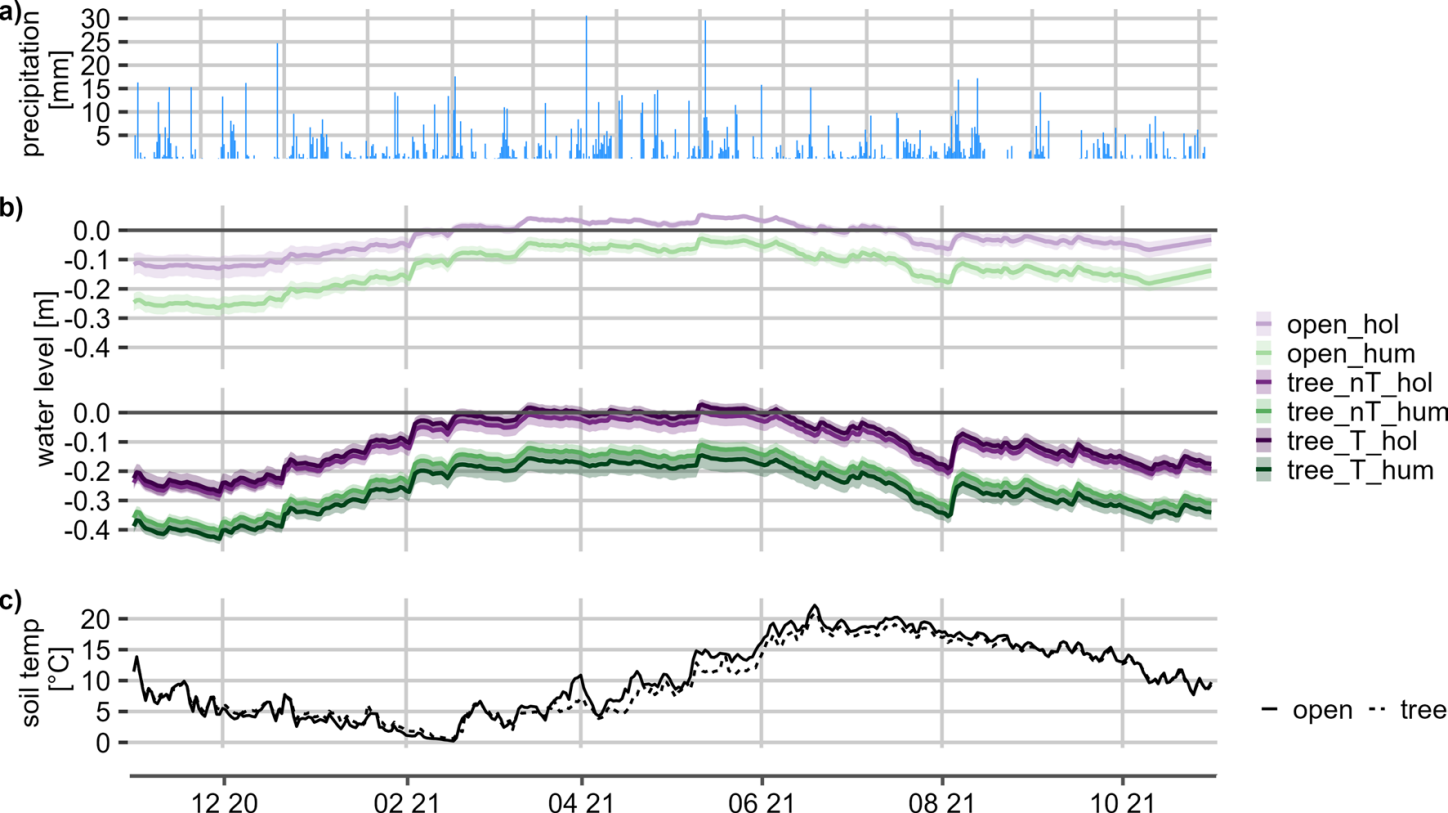
Hydrometeorological data for the measurement period (**a**) daily precipitation for Nienburg (German Climate Service, Nienburg (Weser) (12 km), 2022). (**b**) Water level below surface for each microform (open—open site, tree—birch-covered site, hum—hummock, hol—hollow, nT—distant to tree, T—close to tree), colored ribbons showing standard deviation of the plots, grey line indicates peat surface and (**c**) soil temperature in 5 cm depth at both sites.

At the tree site, WL at hollows distant to trees (nT_hol) stayed just below the surface, while it included some periods of inundation starting from March at hollows below trees (T_hol). This was induced by a more pronounced surface relief below trees (Table 1). The WL below hummocks (hum) was clearly lower (Fig. 1).

In summer, WL at both sites went down, with a stronger decrease at the tree site (Fig. 1). Over the measurement year, WL at the tree site were more fluctuating then at the open site. A distinct rise in WL can be observed after precipitation events.

Soil temperatures in 5 cm depth show the same pattern at both sites, but are slightly higher in summer and slightly lower in winter at the open site. The daily median soil temperatures vary between 0.2–22.2 °C (open site) and 0.6–20.9 °C (tree site). The distribution of microforms varied between the two sites. The tree site had a higher areal contribution of hummocks (61.9 ± 5.5% in total), while the open site was covered with hollows by 56.5 ± 2.2%. The proximity to trees did not influence the proportion of hummocks and hollows at the tree site. DOC in peat pore water at the tree site was almost twice as high as at the open site (Table 1). Additionally, there were slightly higher DOC concentrations below microforms which were located close to a tree. At the tree site, the DOC concentrations were higher below hummocks, which could not be observed at the open site. DOC concentrations below hummocks were higher in the summer than in the winter, while the DOC concentrations below hollows did not show a clear seasonal pattern. Electrical conductivity shows the same pattern as DOC concentrations. The pH was rather constant over the year and marginally higher at the open site, but there were no differences between microforms. At the hummocks, LAI was highest at the open site and lower below trees than distant to trees, with differences in maximum LAI being particularly pronounced. Contrastingly, LAI was lowest at open hollows and highest at hollows below trees, as an opponent of moss cover (Supplementary Tables S1 and S2).

### Measured CH4 fluxes

**Figure 2:**
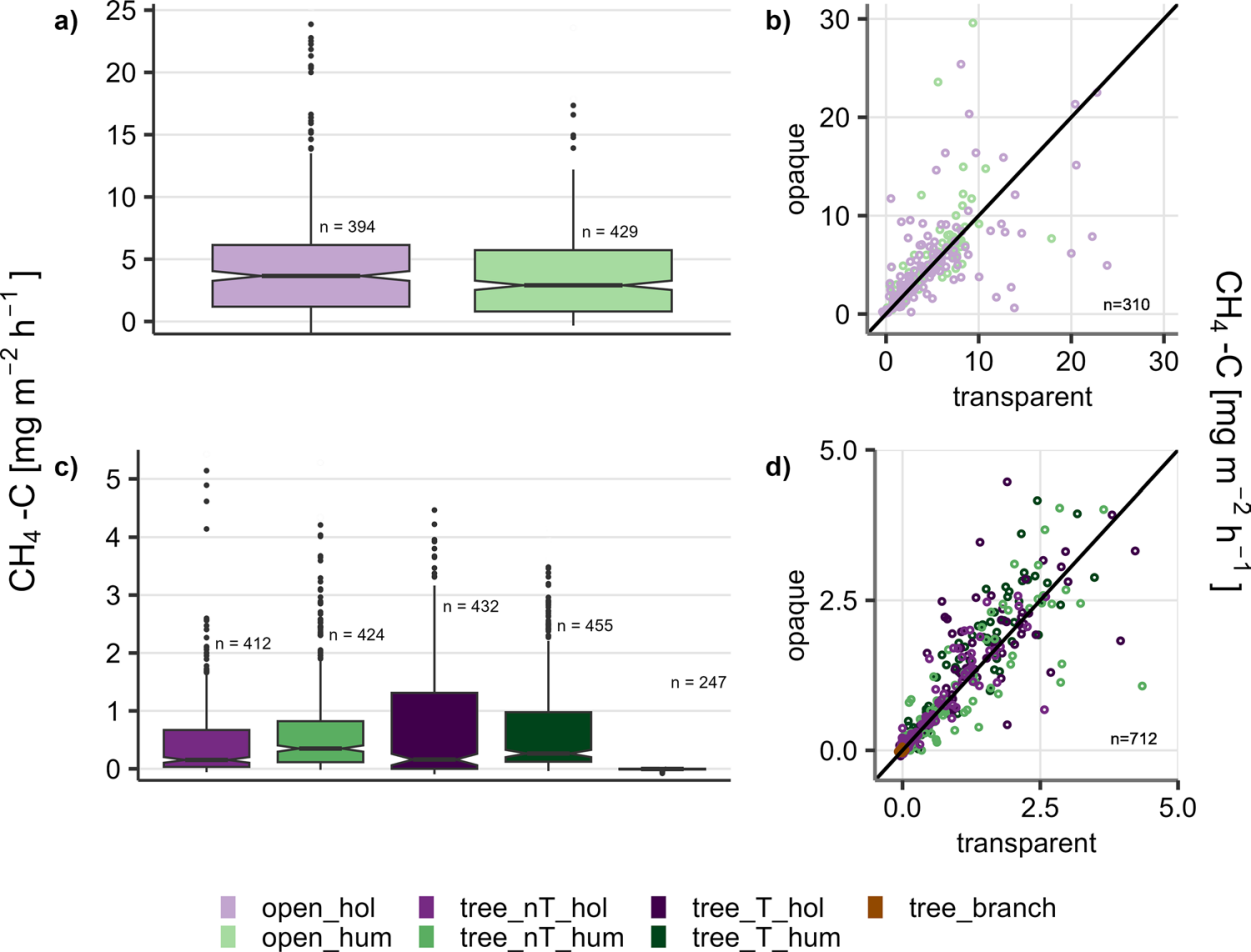
(**a**,**c**) Valid measured fluxes of all microforms, with number of fluxes (n). Boxplots show upper and lower quantile (0.25 and 0.75), black line indicates median. The top 0.5% of fluxes of each microform are not shown and different scales are used for both sites to ensure good comparability and visibility of flux distributions. (**b**,**d**) Paired fluxes from dark and transparent chambers of each microform. Each flux from a transparent measurement was paired with the nearest flux from a dark measurement at the same plot in the time range ± 60 min (this resulted in no pairing in some cases). Black line indicates the 1:1 line.

Measured fluxes ranged between − 1 and 29.6 mg CH_4_-C m^−2^ h^−1^, with the highest values at the open site (Fig. 2). The two highest fluxes occurred at open_hum, followed by open_hol (max. flux 25.4 mg CH_4_-C m^−2^ h^−1^), which also showed the highest median (3.7 mg CH_4_-C m^−2^ h^−1^). At the tree site, highest fluxes were measured at hollows (9.8 mg CH_4_-C m^−2^ h^−1^ (tree_nT_hol) and 8.3 mg CH_4_-C m^−2^ h^−1^ (tree_T_hol)), but highest medians occurred at hummocks (0.35 (tree_nT_hum) and 0.26 mg CH_4_-C m^−2^ h^−1^ (tree_T_hum)) (Fig. 2a,c). Medians at hollows were around half as high (0.16, (tree_nT_hol), 0.16 mg CH_4_-C m^−2^ h^−1^ (tree_T_hol)). Especially at the tree site, hollows showed a number of slightly negative or close to zero fluxes (n = 110, T_hol / n = 73, nT_hol). All microforms showed outliers and the microforms below trees showed a broader range of fluxes between the quantiles, especially the hollows. Overall, the two sites showed clear differences in flux magnitudes, the fluxes from the open site were generally higher than those from the treed site.

A significant difference between microforms (hummocks/hollows) could be shown in most seasons, only for the microforms close to tree in summer and autumn and on the open site in winter, this could not be confirmed.

There was no significant difference in CH_4_ emissions regarding the presence of trees (nT/T).

No differences between opaque and transparent fluxes could be shown for any combination of season and microform. No correlation with PAR was found (Fig. 2b,d).

To add the trees to the balances, *Betula* branches were measured using plant chambers. They showed a small median uptake (− 0.003 ± 0.02 mg CH_4_-C m^−2^ h^−1^), ranging from − 0.08 to 0.06 mg CH_4_-C m^−2^ h^−1^, with the majority of fluxes showing zero or non-detectable fluxes.

### Method validation

The results of the cross validation with observations indicated for all investigated statistical values of model quality, that the environmental parameter-based interpolation methods had a better prediction quality than the linear interpolation. The comparison of Lloyd and Taylor based interpolation and the method additionally including water level (WL + ST) showed that especially in the wettest microform (open_hol) the prediction quality increased clearly when WL was included: R^2^ increased from 0.26 to 0.48, RMSE decreased from 2.44 to 2.08 mg CH_4_-C m^2^ h^−1^, only the bias was lower for Lloyd and Taylor (0.0), but with a higher standard error. Additionally, AIC was clearly higher for this microform. For all other microforms, most results were very similar (Table 2). As one aim of this study is the comparison of all microforms, the WL + ST method was selected. Thus, a more balanced data quality for all microforms was achieved with an overall high interpolation performance. An overview of the prediction quality for the selected WL + ST model is shown in the supplementary material (Fig. S3). Despite the overall increase of bias with increasing flux magnitude, impacts of environmental conditions like water level or temperature on the model bias could not be observed.

**Table 2:** Statistical comparison of all interpolation methods: WL + ST: Non-linear dependency, using water level (WL) and soil temperature (ST), Lloyd–Taylor function and linear interpolation. Displayed values are the mean (RMSE and bias [mg CH_4_-C m^−2^ h^−1^]) and median (R^2^) of the model cross validation with the observations (Number of bootstraps: 1000) and their standard errors. For the methods including environmental parameters, AIC is shown. Bias describes the mean error between predicted and measured values.

microform	WT + ST				Lloyd and Taylor				Linear		
	R^2^	RMSE	Bias	AIC	R^2^	RMSE	Bias	AIC	R^2^	RMSE	Bias
open_hum	0.81 ± 0.2	1.45 ± 0.5	0.11 ± 0.6	− 181	0.81 ± 0.1	1.39 ± 0.4	0.09 ± 0.5	− 185	0.75 ± 0.2	1.77 ± 0.4	0.16 ± 0.8
open_hol	0.48 ± 0.2	2.08 ± 0.4	0.05 ± 0.8	− 108	0.26 ± 0.2	2.44 ± 0.6	0.0 ± 0.9	− 92	0.19 ± 0.3	2.24 ± 0.6	0.55 ± 0.6
tree_nT_hum	0.77 ± 0.1	0.51 ± 0.2	− 0.01 ± 0.2	− 293	0.78 ± 0.1	0.47 ± 0.2	− 0.02 ± 0.2	− 293	0.59 ± 0.2	0.73 ± 0.2	0 ± 0.4
tree_nT_hol	0.55 ± 0.2	0.64 ± 0.4	0.03 ± 0.3	− 254	0.60 ± 0.2	0.61 ± 0.4	0.02 ± 0.3	− 253	0.33 ± 0.2	0.85 ± 0.5	0.06 ± 0.4
tree_T_hum	0.74 ± 0.2	0.49 ± 0.2	0.02 ± 0.2	− 286	0.74 ± 0.2	0.49 ± 0.2	0.01 ± 0.2	− 288	0.51 ± 0.2	0.63 ± 0.2	− 0.02 ± 0.3
tree_T_hol	0.74 ± 0.1	0.54 ± 0.2	0.07 ± 0.2	− 274	0.78 ± 0.1	0.53 ± 0.2	0.06 ± 0.2	− 278	0.67 ± 0.2	0.77 ± 0.2	0.1 ± 0.4

### Flux modelling—intra-annual dynamics

Modelled daily fluxes showed, as well as the measured fluxes, a strong difference between both sites. At the open site, both microforms had constantly higher emissions compared to the tree site. Hummocks showed lower emissions than the hollows in late winter and early spring, but higher emissions during ongoing vegetation period. Modelled maximum emission of hummocks were 0.28 g CH_4_-C m^−2^ d^−1^ on June 19th, compared to 0.23 g CH_4_-C m^−2^ d^−1^ of hollows on June 5th.

**Figure 3:**
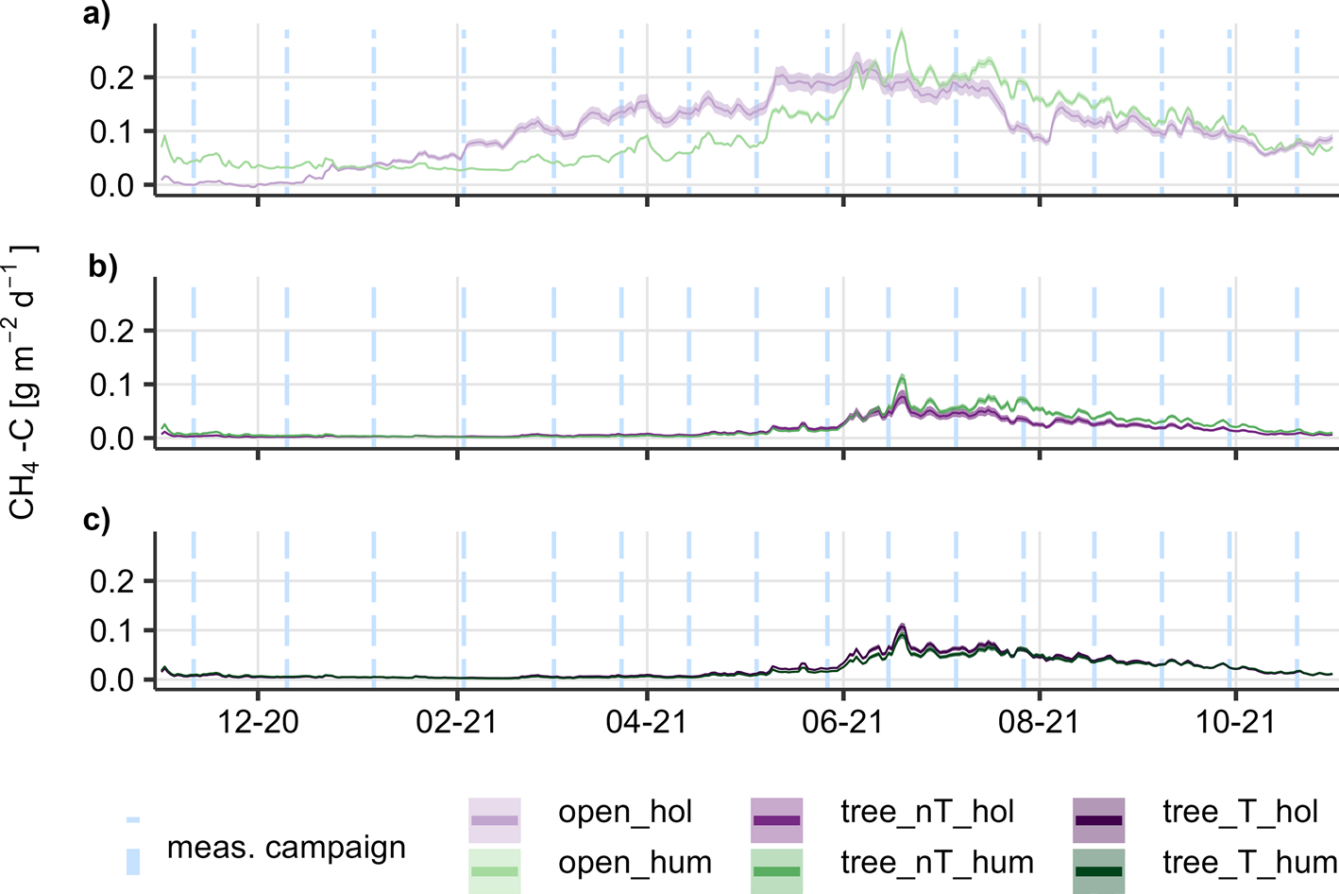
Annual fluxes modelled with soil temperature and water level; suffix hum indicates hummocks, hol indicates hollows, (**a**) open site; (**b**) tree site, distant to tree; (**c**) tree site, close to tree). Blue dot-dashed lines mark measuring campaigns, ribbons mark standard error.

At the tree site, modelled CH_4_ emissions from all microforms were in the same range, with smaller differences between hummocks and hollows. Maximum emissions occurred in June (between 0.08 and 0.11 g CH_4_-C m^−2^ d^−1^ for all microforms) in correspondence to the highest soil temperature. At the nT-microforms, differences between hummocks and hollows in the vegetation period were clearer compared to the T-microforms. At the nT-microforms, hummocks show higher emissions from late June on, while at T-microforms the hollows fluxes stayed slightly higher as the hummocks (Fig. 3).

The annual balances for the birches were calculated with the linear interpolation method. The branch fluxes did not change over the year and stayed close to zero.

### Microforms and methane annual balances

**Figure 4:**
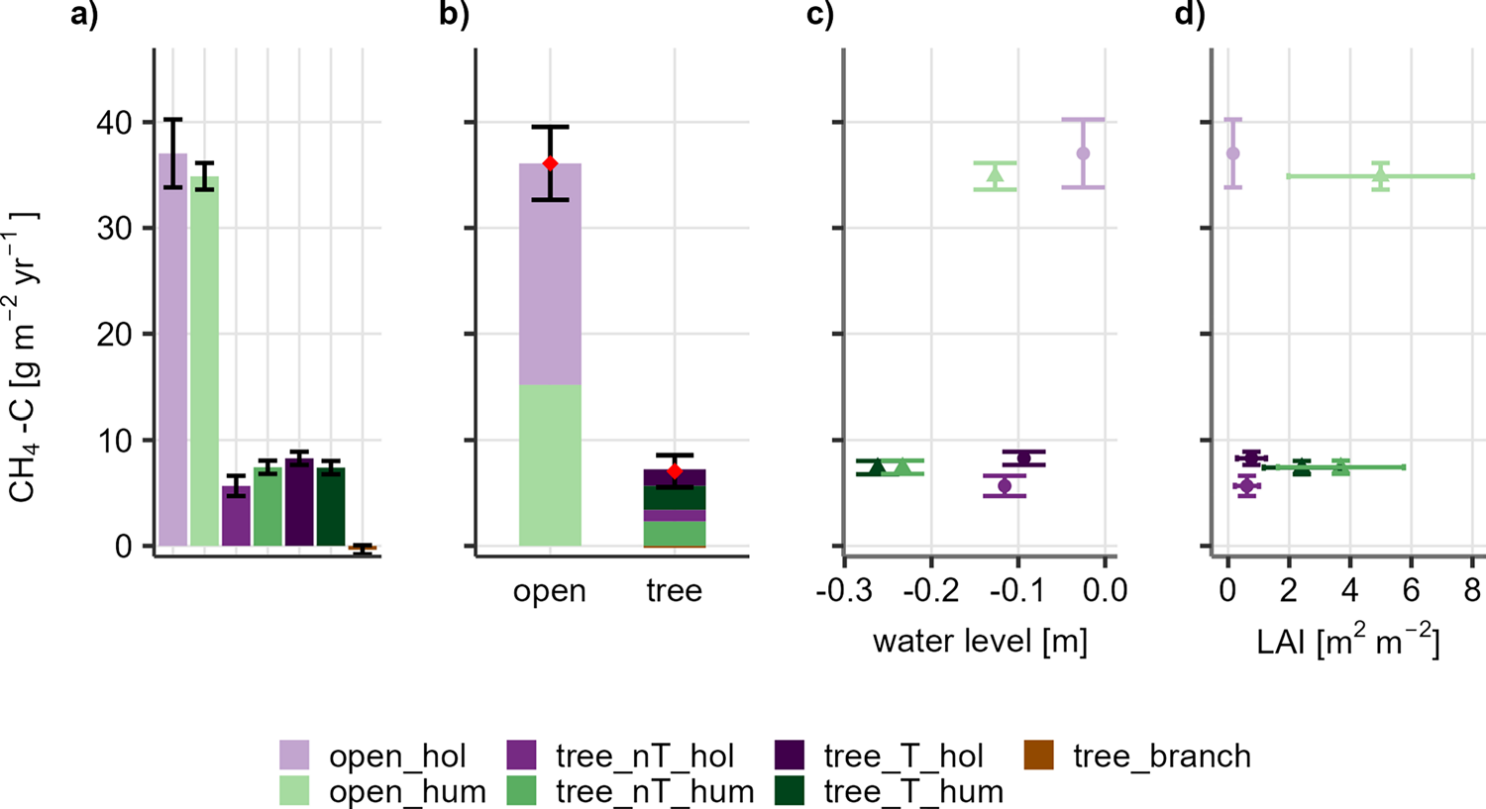
(**a**) Emissions from each microform (CH_4_-C [g m^−2^ year^−1^]) and standard derivation, modelled with soil temperature and water level (only tree_branch was modelled with linear interpolation), (**b**) total annual balances calculated for both sites based on the areal contribution of each microform, (**c**) relationship of methane emissions and mean annual water level across microforms and (**d**) of mean LAI (m^2^ m^−2^) of each microform over the vegetation period (1st March 2021–31st October 2021). Suffix hum indicates hummocks, hol indicates hollows, T indicates the direct proximity to trees, nT indicates distance to trees.

Annual emissions from microforms differed distinctively between the open site and the tree site, with approximately four times higher emissions from the microforms at the open site. Highest emissions per microform originated from hollows at the open site (37 ± 3.2 g CH_4_-C m^−2^ year^−1^). The much lower emissions from the tree site did show different patterns, with hollows having higher emissions close to a tree, but hummocks distant to trees. The highest emissions at the tree site were originating from the T_hol microforms (8.3 ± 0.6 g CH_4_-C m^−2^ year^−1^). The branches contributed only marginally to the annual sums with little or almost no CH_4_ uptake (− 0. ± 0.4 g CH_4_-C m^−2^ year^−1^) (Fig. 4a).

Upscaled emissions per site were found to be 36.1 ± 3.4 g CH_4_-C m^−2^ year^−1^ for the open site and 7.1 ± 1.5 g CH_4_-C m^−2^ year^−1^ for the tree site (Fig. 4b). While at the open site, hollows were emitting the main share (58%), the contribution at the treed site was only 39% of total emissions. The high CH_4_ emissions at the open site corresponded to a high mean WL, with the hummock emissions being slightly lower, despite the high LAI. At the open site, emissions from hummocks were around four times higher than the emissions from hollows at the tree site, but with almost the same mean WL. Hummocks at the tree site, with clearly lower mean WL but higher LAI, had emissions in the same range then the hollows.

Weighted annual mean WL for the sites were − 0.07 m for the open site and − 0.2 m for the tree site. Calculated in CO_2_-equivalents with emission factor 28 based on IPCC^9^, this equals 13.48 and 2.65 t CO_2_-eq.ha^−2^ year^−1^ for the open and the tree site, respectively.

## Discussion

Regarding the **distribution of microforms**, the tree site has a higher share of hummocks compared to the open site. Due to more varying water levels, *Sphagnum* growth is decreasing and vascular plants and shrubs can take over^46^. This leads to an increasing height difference between microforms and a vegetation structure that is more dominated by hummocks, which is very pronounced at the tree site^47^. The high variability in surface structure impeded the determination of the soil surface. Especially hummock plots are heterogenous in their surface height, with differences in one plot up to 42 cm on the tree site (maximum on open site 27 cm). Due to lower *Sphagnum* growth at the tree site, the surface height at hollow plots is showing large unevenness in each plot, especially compared to the hollows at the open site. This surface non-uniformity made it difficult to determine the precise water level, which thus can exhibit large heterogeneity even within one plot.

Many other studies observed, that **CH**_**4**_ **emissions differ between microforms**—in most cases, hollow emissions are higher than hummock emissions^27,29,30^, but Saarnio et al.^48^ and Korrensalo et al.^49^ had rather similar emissions among microforms. They found that under lower water tables the emissions remained similar due to higher LAI of *Eriophorum*. Korrensalo et al.^49^ showed, that even drier microforms with higher LAI had similar fluxes, traced back to microbial processes. Vascular plants can increase peat decomposition^23^, which can be a source of DOC and hereby influence CH_4_ emissions^14,49^. Hommeltenberg^26^ also mentions this as a lagged process, which leads to long-term differences in methane emissions due to peat properties and vegetation. DOC concentrations at the tree site were noticeably higher, especially at below tree-plots, and higher under hummocks then hollows. This might also be responsible for the slightly higher emissions from the hollows close to trees compared to the hollows more distant to trees, despite high spatial proximity. In the case of the hummocks, however, this might be compensated for by the higher *Eriophorum* LAI of hummocks distant to trees.

High variability is common for CH_4_ fluxes and can be correlated to many parameters^8,50^. Surprisingly, we could not find any **diurnal cycles of CH**_**4**_ emissions despite an overall strong influence of *Eriophorum* on CH_4_ fluxes. Differences between transparent and dark fluxes could not be observed and, alike, the PAR reliant CH_4_ release by plants was not reflected in our data, similar as shown by Greenup^20^. This could indicate, that the CH_4_ transport of *Eriophorum* is dominated by molecular diffusion, which is not linked to stomatal aperture. It is contrasting to pressurized flow, which can lead to diel patterns in CH_4_ emissions (e.g. *Phragmites*)^51^. This can be important with regard to existing data sets from projects without transparent chambers. Nevertheless, this information must be used carefully and verified by further research. Additionally, a reaction delay of a few hours may lead to a lack of correlation between fluxes and other parameters^50^. This might be another explanation for the missing direct correlation, especially regarding soil temperature. In our study, high morning fluxes were observed at some campaigns but not being consistent or significant. Overall, however, higher water levels at site level were the prerequisite for higher CH_4_ emissions in our study.

The annual emissions were more than five times higher at the **open site compared to the tree site**. Especially in combination with inundation, CH_4_ emissions can reach high magnitudes as already shown in Abdalla et al.^16^ or Moore et al.^52^. If plots are inundated, CH_4_ cannot be oxidized in the upper peat layer, which is leading to a direct CH_4_ release. The mean water level at the open site was − 0.07 m, with longer periods of inundation, especially at the hollows. Drösler et al.^53^ mark − 0.10 m water table depth as critical for particularly high CH_4_ emissions after rewetting. The high water levels may mainly have caused the high spring and summer emissions, therefore in drier years the methane emissions might tend to be lower. Mean water levels at the tree site were just at the frequently proposed threshold of − 0.20 m for the occurrence of significant CH_4_ emissions^54^. Even if the water level was higher over the summer period, the annual CH_4_ release did not reach the level of the open site. This could also be related to the denser root system in the peat at the tree site, which can enhance the oxygen availability^55^ and thus inhibit CH_4_ production and increase methanotrophy. Regarding the soil profile, higher CH_4_ emissions would have been expected at the tree site, given the higher N- and P-content and lower C:N-ratio^56^. However, these peat characteristics were probably compensated for by other parameters. Additionally, the site was very dry the months before. The low emissions could therefore be supported by a low long-term water saturation, as a delay of over 80 days between full water saturation and CH_4_ production has been observed elsewhere^57^. Another factor that might account for lower emissions from the tree site is the lower *Eriophorum* LAI and, due to shading, even lower in direct proximity to trees. Even though mean soil temperature at the tree site was lower, presumably also caused by shading, this could not explain differences in emissions between the sites.

Even if the differences between microforms within one site were small in term of annual sums, there was a clear pattern in the **intra-annual dynamics of fluxes**, especially at the open site. The hummocks showed higher emissions than the hollows in the vegetation period and the same level (tree sites) or even lower (open site) during the rest of the year. Especially in the vegetation period, *Eriophorum* has been shown to take the major part in CH_4_ release^19,52,57^, which is supported by our data especially from the open site. Roots were found deep in the permanently water saturated peat layer fostering gas transport to the surface. An interaction between vegetation period, plant mediated gas transport and higher soil temperatures in the summer months is indicated in our data. CH_4_ emissions from the hummocks are less dependent on water levels, as shown by higher emissions despite lower water levels from open_hum than from open_hol in summer. Similarly, there were higher emissions from nT_hum at the tree site compared to the hollows there, despite lower water levels.

To add the trees to our balances, we used **birch branch fluxes**. The branches show little to no uptake, and no correlations with environmental parameters. Vainio et al.^44^ measured fluxes in the same range, but mainly CH_4_ release, while Sundqvist et al.^45^ found similar results as in our study, but additionally a slight correlation with PAR. Due to limited resources, we could only measure branch and leaf CH_4_ exchange and gathered no observations on stem emissions, which had also been shown to contribute to total ecosystem fluxes. Vainio et al.^44^ and Pangala et al.^43^ found that birch stem emissions decrease with the height of the stem and age of tree, which would lead to relatively high emission contribution in a very young birch stand as at the tree site. However, in total, contribution of birches to annual CH_4_ budget is reported to be low^44^, Pangala et al.^43^ showed it to be 4.5% (in this case 0.02 g CH_4_-C m^−2^ year^−1^) for a 3-m birch stand on a temperate forested fen.

CH_4_ release via ebullition was explicitly excluded in this study. As ebullition in a similar ecosystems contributed 2–8% of total bog emissions^58,59^, actual emissions might be higher than our reported total site emissions. Based on experience from the field measurements, this has a stronger impact on the wetter sites.

Including soil temperature or soil temperature + water level into the **calculation of annual methane budgets**, decreased bias and RMSE and increased R^2^ compared to the third employed method, the commonly used linear interpolation. However, methods one and two bear the risk of overestimating single flux values, whenever soil temperature or water level exceed the calibration range. Since modelled CH_4_ fluxes at high temperatures showed reasonable values and only data of a few days was extrapolated, possible effects on annual sums are considered negligible. To improve model robustness, we used the median flux per plot and day for calculations, as similarly observed in Juszczak and Augustin^60^, using daily mean, or in Kettunen et al.^27^, who built diurnal classes for their regression models. This can be done to lower the impact of episodic high emissions and seemed to be an adequate solution here to handle the high daily variability of fluxes. Regarding the data from this project, it is of high importance to have multiple measurements over the course of the day, to get a robust data set.

Even though mean annual water level is a good predictor for annual balances^61^, the daily measured value is often not helpful for predictions as further parameters are influencing^52^. For our data, the method including water level showed a better fit to the observations, mainly for the wettest microform. This makes the use of the more flexible water level and soil temperature including model particularly useful in peatland areas that experience strong annual fluctuations due to imperfect rewetting or changed precipitation regimes in times of climate change.

In total, the measured emissions of microforms are in the range of other studies^22,27,57,62^. The main share of CH_4_ emissions from the open site is, due to higher emissions per area and the higher areal contribution, released from the hollows. On the tree site, the hummocks do emit distinct higher amounts of CH_4_. It is indicated, that microforms change parts in CH_4_ release when environmental conditions change.

Using our modelled data, **annual balances** are 36.1 g CH_4_-C m^−2^ year^−1^ for the open site and 7.1 g CH_4_-C m^−2^ year^−1^ for the tree site. In relation to water levels, the results fit to the collection from Abdalla et al.^16^. They are also comparable to the collection of Moore et al.^52^, including data from higher latitudes. Vanselow-Algan et al.^11^ present much higher CH_4_ emissions at a *Sphagnum* site after rewetting, but with a mean water table of 1.2 cm, which is showing the large impact of inundation. Drösler^22^ measured CH_4_ emissions in the same range on near-natural sites, with the emissions from bog heathland and bog shrubs site (10.7 ± 1.6 respectively 5.4 ± 0.5 g CH_4_-C m^−2^ year^−1^) being comparable to the emissions from the tree site in our study. The same study also presents annual emissions of 24.1 ± 1.5 g CH_4_-C m^−2^ year^−1^ for a pristine bog, which is over a third lower than from the open site. There are also many studies representing lower balances for rewetted bogs with comparable vegetation (between 2 – 17 g CH_4_-C m^−2^ year^−1^)^63–66^, which is similar to the emissions from the tree site. Beyer and Höper^67^ are presenting an area with a similar history, but comparably lower annual emissions (22.4 respectively 16.2 g CH_4_-C m^−2^ year^−1^ for a *Sphagnum* respectively *Eriophorum* site). Considering CH_4_, the emissions of both sites are clearly higher compared to unrestored sites after peat extraction (1 g CH_4_-C m^2^)^68^ or active peat extraction sites^59^, and especially in comparison to drained agricultural peatland^69^. Overall, the measured emissions are lower (tree site) respectively within the range (open site) of CH_4_ emission factors for rewetted organic soils in Germany derived by Tiemeyer et al.^61^ (27.9 (14–70) g CH_4_ m^−2^ year^−1^).

In conclusion, our results highlight that the encroachment with birches at this site does not increase CH_4_ release. Emissions are lower, which is probably related to the environmental differences, mainly the lower water tables, that were initially the prerequisite for the development of the tree population. The ongoing birch encroachment and the increasing cover with vascular plants continues to strongly influence conditions, which will have an impact on overall GHG emissions. Together with the CO_2_ balance, this can lead to a complete balance that also includes the changing composition of the microforms. An effect of tree removal on CH_4_ emissions cannot be derived from this data, as effects of many environmental feedbacks are unknown. There are still many former and active peat extraction areas in Europe that need to be put to a suitable subsequent management. Still, agricultural land use or forestry under drained conditions is often planned, consequently leading to strong CO_2_ emissions. It must be the main objective, to reduce CO_2_ emissions in order to mitigate climate change, thus it is of utmost importance to opt for the rewetting of peatlands. Comparably small methane emissions should not be an obstacle to this.

## Methods

### Study area

The two study sites are located in the nature protection area ‘Weißer Graben’ (502 ha) in north-west Germany (52°42′02″N 9°22′05″E). They are part of the Lichtenmoor, which is a bog complex of 2220 ha. Both sites are positioned in the same former peat extraction area, where rewetting (building dams and closing ditches) started in 1986, with ongoing measures up to now. The climate is temperate-oceanic with annual precipitation of 731 mm and annual mean temperature of 9.9 °C (1991–2020, German Climate Service, Nienburg (Weser) (12 km), 2022).

One of the sites is located in the center of the rewetted area and has a high cover of *Sphagnum*-mosses (mainly *S. Cuspidatum*) and *Eriophorum* (mainly *E. vaginatum*)*.* This site represents a close to natural vegetation, with a low density of trees and shrubs and a higher, more constant water level (open site). The other site is around 400 m eastwards, closer to the edge of the rewetted area. The site is dominated by a dense birch population (*Betula pubescens*), with *Eriophorum vaginatum* in the understory, a much lower *Sphagnum* density (mainly *S. Cuspidatum*) and a more varying water level (tree site). Median height of birch trees was 1.67 m with maximum tree height reaching around 4 m. The maximum age of the trees was around 10 years, with a high share of younger trees.

The peat thickness at the open site is around 150 cm, with slightly to moderately decomposed peat down to 110 cm (degree of decomposition of H2 to H5 according to von Post^70^), classified as Ombric Fibric Floatic Histosol^71^. Rooting depth is up to 92 cm. At the tree site, peat thickness is around 100 cm with low decomposed peat only in the upper 30 cm, followed by highly decomposed peat in deeper layers (H7 to H9, von Post^70^), classified as Ombric Sapric Histosol^71^. Rooting intensity is very inhomogeneous, with living roots beyond the first meter.

Peat pH ranges around 3 on both sites, while the C/N-ratio is higher on the open site (51–71 respectively 34–45), due to higher N-content. Phosphorus content is double as high on the tree site and only increased in the first horizons at both sites, while Fe is comparable between the sites, but heterogenous over profile depths. An overview of peat properties can be found in the Supplementary Table S3.

### Field measurements

#### Chamber measurements

Gas exchanges were measured using manual dynamic closed chambers^72^. Chambers (49 cm × 49 cm × 64 cm) were equipped with a fan for air mixing, a pressure equilibration system and openings for temperature probes, which could be closed gastight with rubber seals. The chamber measurements took place from 19th September 2020 to 20th October 2021 every 4 weeks in winter and every 3 weeks during the vegetation period from March to October. The campaigns started before sunrise and continued throughout the day to ideally cover a wide range of soil temperature and solar radiation. Opaque chambers (PVC) and transparent chambers (transparent polycarbonate) were used and at least 4 measurements of both chamber types for all plots were aimed for each campaign. One campaign lasted three days, one day at the open site and two days at the tree site. Depending on the weather, opaque and transparent measurement rounds were conducted one after another, which lead to a certain time difference between transparent and opaque measurements on the same plot (on the open site on average 34 min, on the tree site 59 min). Each measurement lasted for 120–180 s.

The Cavity Ring-Down Spectroscopy Gas-Analyzer GasScouter™ G4301 (Picarro™) was used to measure concentrations of CO_2_, CH_4_ and H_2_O. A data logger (HOBO UX120-006M up to 05/2021, afterwards LabJack U3) was installed, which logged temperature inside the chamber, ambient temperature outside the chamber and, only for measurements with transparent chambers, PAR (LI-COR, Li-190R) outside the chamber.

**Figure 5:**
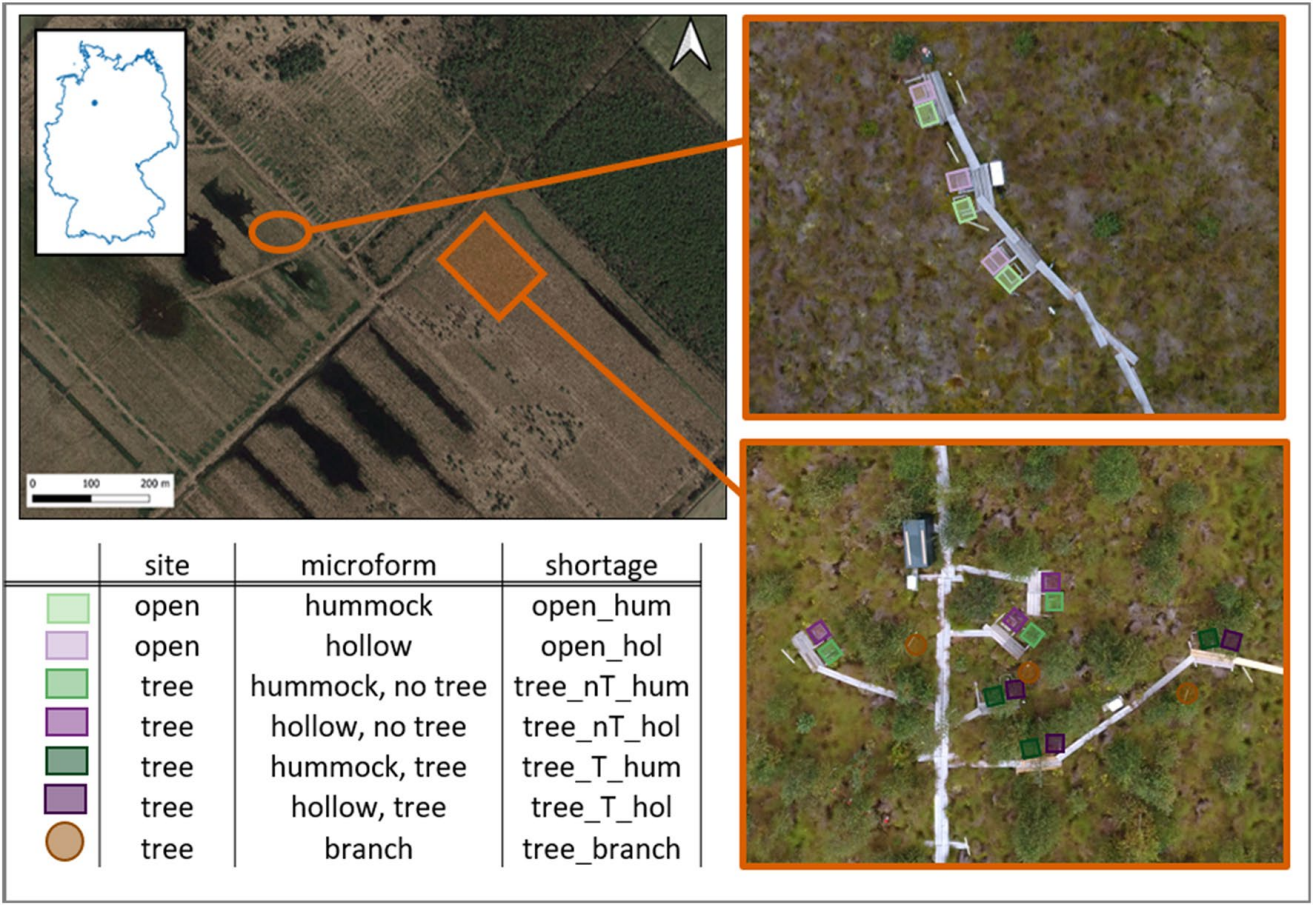
Study area in nature protection area ‘Weißer Graben’ with the open site (upper picture) and the tree site (lower picture). Each rectangle marks one plot, each color represents the three replicates of one microform.

The plots were distributed at each site to capture fluxes from microforms dominated by different plant functional groups. Plots on microforms consisted of a pair each: Hummocks (hum), which are dominated by *Eriophorum vaginatum*, and hollows (hol), dominated by peat mosses. At the tree site, this was again divided into pairs in close proximity to trees (T) and pairs more distant to trees (nT) (Fig. 5). Three replicates for each variant were installed. At each plot a PVC-frame, on which the chamber was tightened to with clamps for each measurement, was permanently installed. The frames were inserted in the peat up to around 10 cm depth, ensuring that they were surrounded by peat, the hummocks were each enclosed by a frame. For each pair of plots a wooden boardwalk was installed on poles to prevent disturbance of the surface. Supplementary to the microform plots, plant chambers were used to measure the gas exchange of birch branches. Therefore, another type of chambers was designed: cylindrical chambers for birch branches (h = 40 cm, r = 9.5 cm). For the plant chambers a fabric shading for the dark measurements was used, with dark fabric inside and white from the outside to prevent heating. In total 21 plots at 7 variants were set up (5 at the tree site and 2 at the open site).

To calculate the precise volume of each chamber, the difference between ground surface and plot frame was measured on 20 points (one horizontal and one opposing transect) every second campaign. The mean of all measurements is used to calculate the corrected chamber volume and this data was also used to determine the height differences within each plot. When plots were flooded or snow covered, the volume of the cover was subtracted from the chamber volume.

#### Water levels

Each pair of plots was equipped with a dip well, which was used for manual water level measurements at each campaign. Measurements of frame and soil surface height relative to the dip well were used to infer the water level below each plot. This way, surface motion caused by oscillation was set into account. Additionally, pressure transducers (Van Essen Instruments, Micro-Diver) were installed at each site to measure continuous water levels spatially representative for both sites. One pressure transducer at each site was located next to the plots and used to interpolate between manual dip well measurements for each plot.

#### Water samples

Borosilicate suction plates (ecoTech GmbH) were installed under each plot 20 cm below the surface of hollows (same absolute level for the hummocks). Suction plates had a pore size of 1 µm, to avoid microbial activity in the sampled water. Water samples were taken approximately once a month for each plot by applying negative pressure via a hose connected to a pump. Two weeks later, water samples were taken from the connected brown glass bottle, which were stored in a dark isolated box. Electric conductivity (EC) and pH were measured in lab. The water samples were analysed for dissolved organic carbon (DOC) (Dimatoc 2000; Dimatec Analysentechnik) in the lab.

#### Biometeorological data

On both sites, meteorological sensors were deployed. At the open site soil temperatures (Campbell Scientific) in three depths (5, 15, 30 cm), air temperature and relative humidity (RH) (HMP155, Vaisala), measured in approximately 1.5 m height were logged in intervals of one minute (CR1000, Campbell Scientific). Logging interval was one minute. At the tree site the meteorological data was logged by the Data Acquisition Module (LI-COR) which was connected to the smartflux-system (LI-COR) and directly saved the data in a compressed 30-min-average file. Air temperature and RH on the tree site were measured in approximately 4 m height, above the canopy, soil temperature was measured between the trees.

#### LAI measurements

Leaf-area index (LAI) was measured for *Eriophorum* spec. and trees 17 times over the vegetation period. Grass blades were counted in-situ with a 5 × 5 cm frame. This was done once for each section with different leaf density or height. For each small frame, the height of 30 randomly chosen blades were measured (only the green, i.e. photosynthetically active part). The LAI was calculated as average height multiplied with the leaf area per length. The share of total plot area was estimated and upscaled. Measurements were repeated every fortnight in times where phenological state was assumed to be highly variable (spring and fall). In addition, an intensive campaign was carried out to determine LAI with higher precision, using three frames per section for hummocks and counting all grass blades in hollows, respectively. The highest measured LAI was assumed to be maximum LAI.

Tree LAI was measured using the Plant Canopy Analyzer (LAI-2200C; LI-COR), following the same biweekly rhythm as the grass LAI. Tree LAI is including birch (*Betula pubescens*) and pine (*Pinus sylvestris*), and the share of the pine was ignored. Data from tree inventories showed that *Pinus sylvestris* accounts for only 7% of tree individuals, so in combination with the overall lower LAI of conifers, a very little contribution to total LAI was assumed.

To determine the area of green leaves in the plant chamber, the leaves of birches were counted during each campaign. To determine the mean area per leaf, a leaf sample campaign for birches took place in summer 2021. Three branches of five trees were sampled, scanned, and the mean leaf size was calculated. Additionally, to obtain length related leaf area [m^2^ cm^−1^] for *Eriophorum*, ten grass blades for each replicate were sampled and scanned. For computing the leaf area from scanned images, the image processing program 'ImageJ' version 1.53c^73^ was used.

#### Microform distribution

For each site the distribution of microforms (hollow, hummock, close to/distant to tree) was determined. This was done for ten (treed site) and five (open site) transects (10 m), which were laid out across the area of both sites. The aim was to cross the area selected for balancing as evenly as possible without interference from project installations in the transects. At each cm the prevailing structure was noted. Microforms at the tree site were divided again into close to (T) and distant to tree (nT), depending on the respective distance to the next tree. The trees were divided into three size classes and a maximum distance was determined for each class to subdivide nT and T (0.5 m, 1 m, 1.5 m). This data was used to calculate the areal share of each microform and its standard derivation.

### Flux calculation

All analyses were conducted using R software (version 4.2.2)^74^.

Raw data for each flux was merged with the associated environmental data. As PAR was measured outside the chambers, it was reduced by 5% for the flux calculations, to account for PAR reduction due to material absorption. As dry-values are supplied, no water vapor correction was necessary. The first 10% of values of each flux measurement were removed to avoid artifacts due to chamber placement. We adopted a method previously used for CO_2_ flux calculations in Oestmann et al.^62^ using a linear regression. Depending on solar declination, a moving window ranging from 40 s (summer) to 60 s (winter) was used for flux calculation. Each window with R^2^ < 0.8, a temperature change > 1.5 °C or > 10% PAR change was excluded. The window with the highest coefficient of determination (R^2^) was chosen for flux calculation. If the increase of CH4 was lower than 3% (0.03) of the mean concentration, the R^2^ criteria was not applied, to not discard low fluxes. All fluxes are additionally manually controlled and erroneous fluxes were excluded (e.g. ebullition). Minimum detectable flux (MDF) was calculated as described by GSS^75^, using 0.8 ppb for analyzer precision. In total, 346 fluxes were detected to be below MDF, most of them branch measurements (42%) and hollows on the tree site (29 respectively 22%). Fluxes were kept in the dataset, to avoid a biased modification of the dataset. The dataset was additionally checked for high morning fluxes, which seemed to be caused by stable conditions. A relatively cautious approach was chosen, selecting fluxes starting above 3 ppm, represent the highest flux of the day from the plot and were the first measurements of the day before 8 am. In total, 13 fluxes were removed by this method. No indications of non-linear emission behavior during the selected windows could be observed. The total amount of valid fluxes was 2817.

### Statistics

To test the data for significant differences between dark and transparent fluxes, the non-parametric Mann–Whitney-*U*-test (package: ‘psych’, version 2.3.9^76^) was used for each combination of microform and season. The Null-Hypothesis indicates that there are significant differences between dark and transparent fluxes. The seasons were divided by month (December–February: Winter, March–May: Spring, June–August: Summer, September–November: Autumn). Significance level of 5% (p = 0.05) was applied. The Mann–Whitney-*U*-test was also used to test for differences between the microforms for each season and to test if the proximity to trees was responsible for significant flux differences.

### Annual balances

To calculate annual balances of all sites, three modelling methods were compared to find the solution best representing the observations and utilizing as much information from environmental covariates as necessary. A method including water level and soil temperature (WL + ST)^27,29^ was compared with a method using only soil temperature. Additionally, a linear interpolation method was used for an estimation without the impact of environmental parameters.

Since a significant difference between opaque and transparent fluxes could be negated in the section before (see above), all measurements were pooled together for further calculations. Fluxes showed a strong exponential relationship with soil temperature when the median flux and the median soil temperature per measurement campaign and plot were used (Supplementary Fig. S1). Thus, the data set was prepared for further analysis accordingly. This resulted in three temperature and flux pairs per campaign for each microform which were used in all modelling methods to ensure comparability of results. To calculate balances for a full year, data was used from 1st November 2020 to 31st October 2021, which equals 18 campaigns. In total, 2555 fluxes were used to build 310 median values for the calculations of annual balances. An overview of all determined parameter can be found in Supplementary Table S4.

(a) Lloyd and Taylor based interpolation

To calculate annual emissions on a soil temperature-based approach, Lloyd and Taylor equation was used^24,77^. Therefore, data was fitted with the temperature response function of Lloyd and Taylor (1994)^77^.

1 $${CH}_{4}= {R}_{ref}* {e}^{{e}_{0}*\left((1/Tref-{T}_{0})-(1/{TS}_{5}-{T}_{0})\right)}$$

TS_5_ is soil temperature (K) in 5 cm depth, T_0_ is a constant (227.13°K), e_0_ is individual ecosystem sensitivity and R_ref_ the individual respiration rate at reference temperature T_ref_ (283.15°K). Rref and e0 were estimated individually by fitting a non-linear least-squares model (nls) (R-package *stats*^74^) for each microform.

Individual determined e_0_ and R_ref_ was used in combination with the median of soil temperature per day to calculate the daily emissions per microform. This data was summed up to obtain annual balances. To get the SE of each site, data was bootstrapped 2000 times by randomly sampling fluxes with replacement, and SE of the results was taken as the uncertainty of the balances.

(b) Water level and soil temperature (WL + ST)

To include a further environmental parameter, a method combining water level and soil temperature was used (WL + ST). Therefore the method introduced in Laine et al.^29^ was applied to the median data set used before.

2 $${CH}_{4}=(c+dWL)({\text{exp}}\left(b{TS}_{5}\right))$$

WL is the individual water level (m) per measurement day for each plot as described above, TS_5_ is soil temperature in 5 cm (°C) depth and b, c and d are parameters, individually determined for each microform. The parameters were estimated individually for each microform using a nls-model (R package stats^74^). Median soil temperature of each site was combined with plot individual WL to calculate daily fluxes. The summed-up data is used as the annual emission estimates. To obtain the SE, data is bootstrapped 2000 times by randomly sampling fluxes with replacement, and the SE was taken for the uncertainty of the balances.

(c) Linear interpolation

Linear interpolation was applied as described in Günther et al.^78^ and adjusted as described in Oestmann et al.^62^. The result is a combination of jackknife and bootstrapping. One of three daily median fluxes of each microform and campaign is selected randomly to build 2000 random time series. For those timeseries, balances were calculated using linear interpolation, leaving out one campaign day at each run. The reported annual emissions represent the median of all jackknife balances. SE is generated over the 2000 bootstraps.

(d) Validation of interpolation method

As there was no correlation with environmental parameter for the branch fluxes, they were modelled with linear interpolation. For the microforms, all methods were tested and compared for their predictive quality over the entire measurement period using the cross-validation method. Therefore, each dataset was divided into a training-dataset containing 80% of the used median fluxes and a test-dataset with the remaining 20%. The data was sampled randomly (using R-package *sample*^74^). The models were calculated as described in 4(a)–(c) with the train-dataset. Fluxes were predicted for the test-datasets and R^2^, RMSE and bias for the predicted fluxes from the test data set and the measured fluxes from the train data set were calculated. This was bootstrapped 1000 times. The same procedure was done for the linear interpolated daily fluxes, which then were compared with the fluxes from the test data.

R^2^ (median), RMSE (mean) and bias (mean) of all datasets were used to compare model capabilities to predict fluxes. The bias was calculated as the mean difference between predicted and measured fluxes, to approve whether the models tend to over-respectively underestimate fluxes.

Because Nonlinear Least Square—models (Package: nlstools, version 2.0^79^) were used for WL + ST and Lloyd and Taylor, the AIC-values could be compared to each other. That was not conducted for the linear interpolation approach.

(e) Annual balances per microform and site

Annual balances were calculated for each microform at first, then multiplied with the areal contribution of each microform on both sites. This gave microform individual annual sums, which were summed up to site related annual fluxes. To get total SE, Gaussian error propagation was used to combine SE of areal contribution and of annual emissions of microforms respectively sites.

Tree fluxes were multiplied with changing LAI over the course of the year, to get the contributions per area. An overview of the results of all methods can be found in Supplementary Fig. S1.

### Supplementary Information

Supplementary Information.

## Data Availability

Data will be made available on request. Please contact Arndt Piayda (arndt.piayda@thuenen.de) for further information.
