# Supplementary material for: Effects of birch encroachment, water table and vegetation on methane emissions from peatland microforms in a rewetted bog
Source: Sci Rep. 2024 Jan 30;14:2533. doi: 10.1038/s41598-024-52349-0 (PMC10828379; doi:10.1038/s41598-024-52349-0)
Supplement: Supplementary file 1 — Supplementary Information. [file 41598_2024_52349_MOESM1_ESM.pdf]

Supplementary Table S1. Vegetation survey of each plot on the field sites in Lichtenmoor (2020-08-26). Numbers denote coverage in %. Vegetation survey was supported by Amanda Grobe.

	open						tree_nT						tree_T					
**plot**	hol_1	hum_1	hol_2	hum_2	hol_3	hum_3	hol_1	hum_1	hol_2	hum_2	hol_3	hum_3	hol_1	hum_1	hol_2	hum_2	hol_3	hum_3
**total**	80	100	100	30	100	100	99	80	95	100	100	100	50	90	90	45	80	45
vascular plants	4	75	5	25	3	20	5	70	8	80	15	75	1	60	30	40	1	45
mosses	80	50	100	15	100	80	99	50	95	75	90	60	50	10	80	20	80	25
peat								2	5				2			25		
water																		
litter	25	20	3	70	1	20	2	20	1	20	10	25	30	40	15	30	20	55
**mosses**																		
Sphagnum cuspidatum	80	50	100	15	100	80	97	5	94		90	60	50	1	65		80	5
Sphagnum fallax											1							
Sphagnum fimbriatum									1	73			1			1		5
Sphagnum palustre														4				
liverwort										1								
other mosses						1	2	45	1	1	5	1	20	5	15	20	0.5	15
**vascular plants**																		
Betula pubescens						1	4		1			0.5	0.5	1	1			
Dryopteris carthusiana						0.5		10			0.5		0.5	10		1		5
Eriophorum angustifolium	4		5		2												0.5	
Eriophorum vaginatum		75	0.5	25	1	20	1	60	8	80	5	8	0.5	50	30	40	0.5	40
Pinus sylvestris		0.5																
Seedlings												0.5					0.5	
**mushrooms**																		
small mushroom	1		3		1													

Supplementary Table S2. Vegetation survey of each plot on the field sites in Lichtenmoor (2021-12-02). Numbers denote coverage in %. Vegetation survey was supported by Laura Panitz.

	open						tree_nT						tree					
**plot**	hol_1	hum_1	hol_2	hum_2	hol_3	hum_3	hol_1	hum_1	hol_2	hum_2	hol_3	hum_3	hol_1	hum_1	hol_2	hum_2	hol_3	hum_3
**total**	85	95	100	70	100	95	100	95	99	100	100	99	99	85	100	90	100	90
vascular plants	5	65	10	40	10	75	10	60	40	45	30	65	30	80	70	55	35	20
mosses	85	40	100	30	100	50	95	50	85	60	95	40	85	15	80	50	95	25
peat	5	1		5				1	1	1		1	5	15		10		10
water	10	1		5		5		0.5										
litter	5	15	1	25	1		25	20	10	15	10	10	15	20	20	20	30	40
**Mosses**																		
Sphagnum cuspidatum	55	40	90	30	100	45	95	10	75		90	30	75	5	70	20	95	10
Sphagnum fallax																		
Sphagnum fimbriatum									10	45								5
Sphagnum palustre														5				
Polytrichum famosum				0.5		1	0.5	30		5		10	5	1	10	5		5
other mosses										5								
Sphagnum spec.	30		10			5	1	10	1	5	5	1	0.5	5	1	25	1	1
**Vascular plants**																		
Betula pubescens							0.5	0.5										
Dryopteris carthusiana				0.5		1		10		0.5		0.5		0.5		0.5		
Eriophorum angustifolium	5	0.5	5		5	0.5											5	
Eriophorum vaginatum	0.5	40	5	40	5	75	10	50	40	45	30	65	30	80	70	55	30	20
Molinia caerulea												2						
algae				0.5														
**Mushrooms**																		
brown mushroom										0.5		0.5					5	
slime mold				1									0.5			0.5		5

Supplementary Table S3: Peat properties and peat chemistry of the soil horizons in the first meter at both sites.

location	horizon depth	von Post	TN	TC	C:N	pH	P	Fe
	[cm]		[g kg^{-1}]	[g kg^{-1}]			[mg kg^{-1}]	[mg kg^{-1}]
open site	0-11	H2	8.29	505.97	61	3.3	61.60	25.05
	11-56	H3	6.84	514.93	75	3.2	37.21	13.40
	56-68	H4	10.80	548.52	51	2.9	4.90	95.83
	68-92	H5	8.88	533.62	60	3	3.18	44.24
	92-110	H5	8.67	537.85	62	3	--	25.82
tree site								
	0-30	H2	12.27	510.13	42	3.2	124.31	21.69
	30-78	H7	12.35	550.49	45	3	5.54	95.67
	78-100	H8	17.23	593.57	34	3.1	--	81.24

Supplementary Figure S1

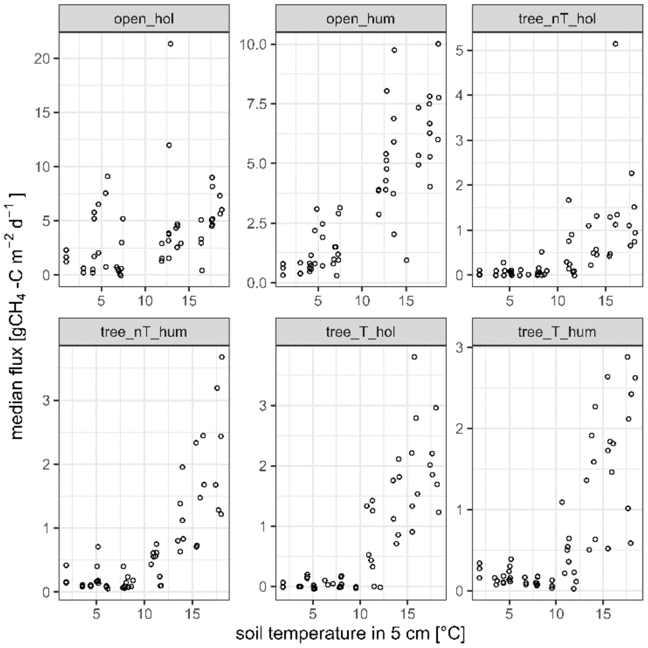

Supplementary Figure S1. Daily median CH4 fluxes of all sites (consisting of 3 replicates each) plotted against median daily soil temperature.

Supplementary Figure S2

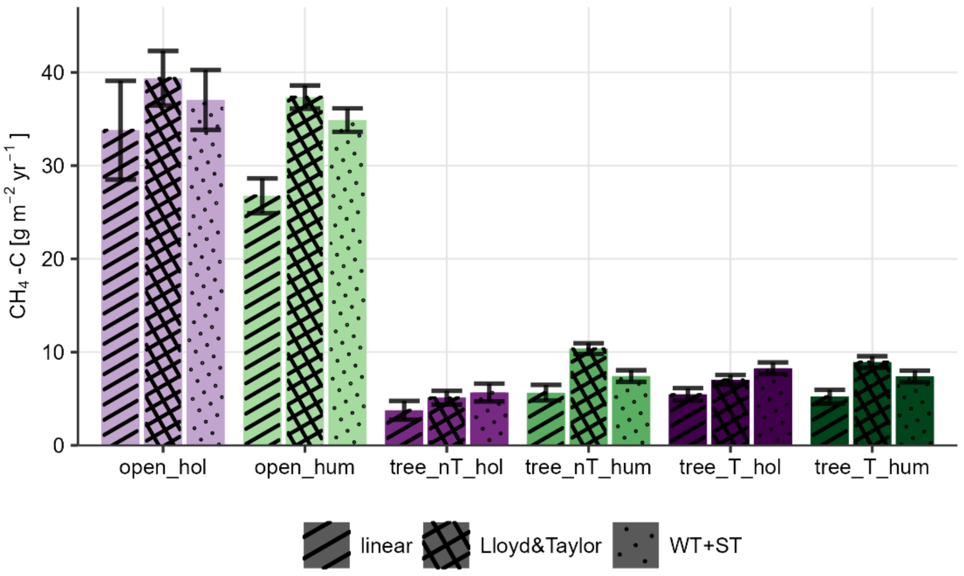

Supplementary Figure S2. Annual sums of each microform. Each pattern represents one method: linear interpolation (linear, stripes), Lloyd & Taylor based interpolation (Lloyd&Taylor, chequered) and soil temperature and water table including interpolation (WT + ST, dotted).

Supplementary Table S4: Determined model parameter for each microform (R_{ref} & e_0: Lloyd & Taylor; c,d,b: Water tabler and soil temperature related model)

microform	R_{ref}	e_0	c	d	b
open_hum	0.0623	409	0.0409	0.0409	0.107
open_hol	0.0829	265	0.0805	0.637	0.0395
tree_nT_hum	0.0084	412	0.00118	-0.00134	0.203
tree_nT_hol	0.00975	588	0.00271	0.0067	0.166
tree_T_hum	0.0135	791	0.00199	-0.00146	0.172
tree_T_hol	0.0146	626	0.00299	0.00358	0.171

Supplementary Figure S3

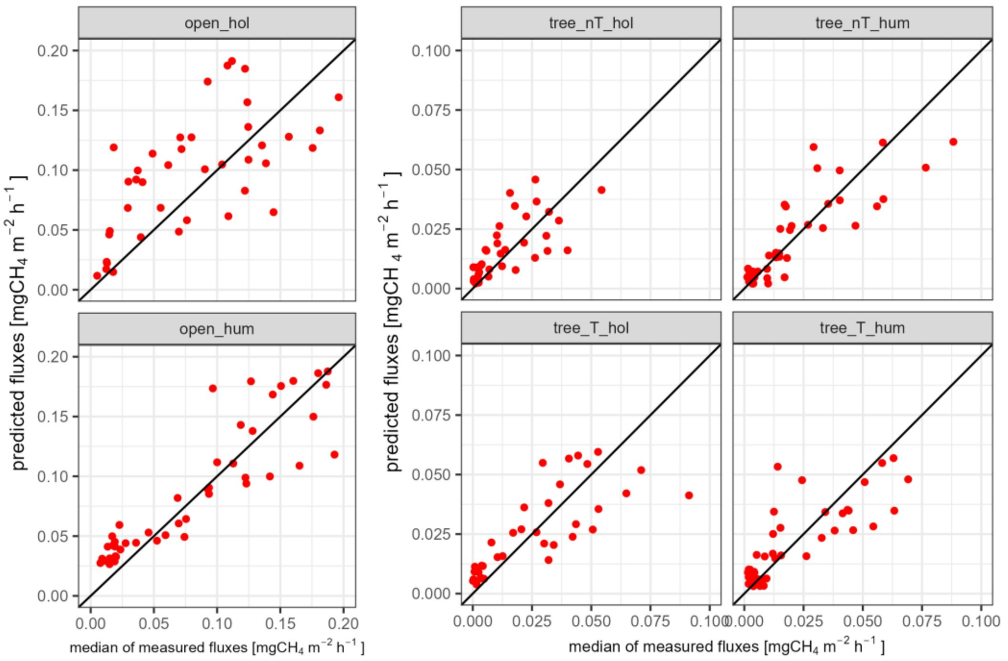

Supplementary Figure S3. Daily median CH_4 fluxes plotted against predicted fluxes (using water table and soil temperature).
